# Nodular Thyroid Disease in the Era of Precision Medicine

**DOI:** 10.3389/fendo.2019.00907

**Published:** 2020-01-23

**Authors:** Dario Tumino, Giorgio Grani, Marta Di Stefano, Maria Di Mauro, Maria Scutari, Teresa Rago, Laura Fugazzola, Maria Grazia Castagna, Fabio Maino

**Affiliations:** ^1^Endocrinology Unit, Department of Clinical and Experimental Medicine, Garibaldi-Nesima Medical Center, University of Catania, Catania, Italy; ^2^Department of Translational and Precision Medicine, Sapienza University of Rome, Rome, Italy; ^3^Division of Endocrine and Metabolic Diseases, Department of Clinical Sciences and Community Health, IRCCS Istituto Auxologico Italiano, Università degli Studi di Milano, Milan, Italy; ^4^Department of Clinical and Experimental Medicine, University of Messina, Messina, Italy; ^5^Endocrinology Unit, Department of Clinical and Experimental Medicine, University of Pisa, Pisa, Italy; ^6^Department of Medical, Surgical and Neurological Sciences, University of Siena, Siena, Italy

**Keywords:** microcarcinoma, thyroid nodule, ultrasound, mini invasive treatment, molecular testing

## Abstract

Management of thyroid nodules in the era of precision medicine is continuously changing. Neck ultrasound plays a pivotal role in the diagnosis and several ultrasound stratification systems have been proposed in order to predict malignancy and help clinicians in therapeutic and follow-up decision. Ultrasound elastosonography is another powerful diagnostic technique and can be an added value to stratify the risk of malignancy of thyroid nodules. Moreover, the development of new techniques in the era of “Deep Learning,” has led to a creation of machine-learning algorithms based on ultrasound examinations that showed similar accuracy to that obtained by expert radiologists. Despite new technologies in thyroid imaging, diagnostic surgery in 50–70% of patients with indeterminate cytology is still performed. Molecular tests can increase accuracy in diagnosis when performed on “indeterminate” nodules. However, the more updated tools that can be used to this purpose in order to “rule out” (Afirma GSC) or “rule in” (Thyroseq v3) malignancy, have a main limitation: the high costs. In the last years various image-guided procedures have been proposed as alternative and less invasive approaches to surgery for symptomatic thyroid nodules. These minimally invasive techniques (laser and radio-frequency ablation, high intensity focused ultrasound and percutaneous microwave ablation) results in nodule shrinkage and improvement of local symptoms, with a lower risk of complications and minor costs compared to surgery. Finally, ultrasound-guided ablation therapy was introduced with promising results as a feasible treatment for low-risk papillary thyroid microcarcinoma or cervical lymph node metastases.

## Introduction

One of the main clinical challenge in endocrine clinical practice is certainly the management of thyroid nodules disease. During the last years, new technologies have been developed and new diagnostic and therapeutic approaches have been introduced to guide clinician through the diagnosis, follow-up and therapeutic decision. This review will provide an evidence-based summary of the optimal approach to the management of thyroid nodules.

## The Key Role of Ultrasound

The prevalence of nodular thyroid disease in the general population is high, reaching 60% according to ultrasound and autopsy findings (1–3), though the incidence of malignancy is relatively low, ranging 1.6% and 12% (4, 5). Thyroid ultrasonography (US) is the primary tool used for the diagnosis and the initial cancer risk stratification of thyroid nodules. Currently, it guides decision making for fine-needle aspiration biopsy (FNA), the timing of subsequent clinical evaluations during long-term follow-up (6), and the eligibility for active surveillance of suspicious nodules (7). A complete report should include a description of the whole thyroid parenchyma, nodule location, size, and sonographic features, and assessment of the lymph nodes in the neck (8–10). The US features that should be evaluated for each nodule are: echogenicity, composition (solid, cystic, mixed), margins, calcifications or other hyperechoic foci, shape, and relations with the thyroid capsule (11, 12). Ultrasound patterns associated with malignancy include: hypoechogenicity, infiltrative, irregular, or lobulated margins, micro-calcifications, taller-than-wide shape, absence of a halo. However, none of these single US pattern have sensitivity, specificity and accuracy high enough to be considered predictive for malignancy (11, 13, 14). The combination of US patterns leads to a higher specificity, but it associates to a lower sensitivity (15). Finally, it is worth to mention that the evaluation of these US features is characterized by a high interobserver variability (16, 17). In the last decade, to improve standardization of thyroid ultrasound reporting, the guidelines of the American Association of Clinical Endocrinologists/American College of Endocrinology/Associazione Medici Endocrinologi (AACE/ACE/AME) (3), the 2015 guidelines of the American Thyroid Association (ATA) (18), the guidelines of the European Thyroid Association (ETA; EU-TIRADS, European Thyroid Imaging Reporting and Data System) (8), the American College of Radiology (ACR) TIRADS (9), and the Korean Society of Thyroid Radiology's K-TIRADS system, have proposed risk stratification systems with the goal of detecting nodule at greatest risk for malignancy and then to recommend graduated size cut-offs for FNA cytology (19) (Table 1). All these risk-stratification systems are similar, but there are some differences: the endocrinological societies' systems are based on recognition of patterns, while ACR TIRADS is score-based, considering 5 US features and their sum to obtain the final classification of the nodule. Furthermore, the weight of each sonographic feature varies across various systems (e.g., echogenicity being the most important one for EU-TIRADS, and composition for K-TIRADS), and the size threshold to recommend FNA is different, too. Some of the systems have been validated in multicenter studies (20–22). Independent comparison studies (usually involving 2–3 of the systems) were mostly retrospective (23–28). Two prospective Italian studies compared the systems developed by the British Thyroid Association, the ATA, and the AACE/ACE/AME (29), or the ATA and the ETA (30) and found no significant differences between overall diagnostic accuracy. A recent study comparing the main five systems endorsed by international societies, found that four of the five (AACE/ACE/AME, ATA, ACR-, and EU-TIRADS) showed a significant diagnostic value (31). The ACR TIRADS, which classified over half of the requested biopsies as unnecessary, with a negative predictive value of 97.8%, showed the best overall performance (31). To reproduce these results in the real clinical practice, an essential prerequisite is the adoption of a uniform language and definition of suspicious features (10). Classification of thyroid nodules using any of the five classification systems results in higher interobserver agreement than evaluation of single suspicious features, and identification of nodules needing biopsy has an almost perfect agreement (32). However, a specific “training by consensus” involving joint evaluation of images can improve the reproducibility for all classifications (with significant improvements for ATA, K-TIRADS, and EU-TIRADS systems), even for trained clinicians with similar experience (32). In recent years, the useful of ultrasound patterns to stratify the risk of malignancy of indeterminate thyroid nodules, has also been evaluated (33–35). Sonographic patterns were associated with different rate of malignancy suggesting that these systems are also able to stratify the risk of malignancy in the subgroup of cytologically indeterminate thyroid. These preliminary data suggest that sonographic patterns would be useful not only to guide FNAC, but also to personalize management after an indeterminate cytological results. Recently, software applications performing automated image analysis were also proposed to extract quantitative parameters using a variety of mathematical methods. These approaches may be the basis for computer-aided diagnosis (CAD) systems to yield an automated “second opinion” (36). According to some evidence, thyroid CADs based on artificial intelligence may further improve diagnostic performance and reliability (37). The use of thyroid CAD to differentiate malignant from benign nodules showed accuracy similar to that obtained by an expert radiologist (38, 39) and may reduce intra- and inter-observer variability, that however, still remains (38). Ultrasound elastography (USE) has emerged as an additional tool in combination with B-Mode Ultrasound (US) for thyroid nodules work-up. It is a non-invasive, cost-effective, dynamic diagnostic method for the measurement of tissues elasticity (40, 41). Malignant lesions tend to be harder and firmer than the normal thyroid parenchyma or benign lesions, related to fibrosis and higher expression of Galectin-3 and Fibronectin-1 (41–43), suggesting that elastography can be useful to distinguish between benign and malignant thyroid nodule (43, 44). There are two main elastography techniques to quantify thyroid nodules stiffness currently in clinical use: strain elastography (SE), which evaluates the degree of tissue deformation induced by manual compression or acoustic forces, and in which tissue deformation is parallel to the direction of the force; and shear wave elastography (SWE), in which a push beam is created and tissue displacement is perpendicular to the direction of the force (45, 46). Many studies and meta-analyses have identified for USE some limitations and confounding factors, including nodule features (calcifications, cystic components, size, position), the operator expertise, artifacts such as carotid artery pulsation, coexistent systemic, or thyroid diseases (Hashimoto's thyroiditis, acromegaly, previous thermal ablation or radiofrequency on thyroid nodule) and pathological type of thyroid cancer (40, 41, 47–71). Therefore, USE should be performed in selected thyroid nodules by qualified operators using objective criteria provided by elastographic machines. Two clinical practice guidelines include recommendation on thyroid USE. The 2015 ATA guidelines (18) reported that USE may be a helpful tool for preoperative risk assessment in patients, although it cannot be universally recommended. The 2016 AACE/ACE/AME guidelines (3) reported that USE data are complementary to gray-scale findings, especially in nodules with indeterminate US or cytological findings. Moreover, other specialized guidelines specific for USE [European Federation of Societies for Ultrasound in Medicine and Biology (EFSUMB) (72) and World Federation of Societies for Ultrasound in Medicine and Biology (WFSUMB) guidelines (50)], provide an adequate description of the technique and its reproducibility, results and limitations. Although many reports have demonstrated that USE performed the same or better than the gray-scale US (40, 41), its diagnostic efficacy is still controversial (73). In clinical practice USE is usually performed as a complementary tool to conventional US, as the combination of the two techniques proved to have higher sensitivity (74). Recently, some studies evaluated the potential role of elastography in non-diagnostic or indeterminate nodules (43, 75), even if conventional US also has been shown to display good diagnostic results (37, 76). Utility of USE has also been explored in many studies (40, 54, 77, 78) but therefore, other authors failed to demonstrate the diagnostic utility in indeterminate nodules (75, 79) and a meta-analysis of eight studies demonstrated there was a great variability of both sensitivity and specificity in USE, with pooled estimates of 69 and 75%, respectively (77). Further studies are required concerning the supplementary role of elastography in the risk stratification of thyroid nodules.

**Table 1:** An overview of the standardized thyroid nodule US scoring systems proposed or endorsed by international practice guidelines.

**Risk score**	**AACE/AME/ACE**	**ATA**	**EU-TIRADS**	**K-TIRADS**
Suspicious US features	Marked hypoechogenicity Spiculated or lobulated margins Microcalcifications Taller-than-wide shape Extrathyroidal growth Pathologic adenopathy	Irregular margins (infiltrative, microlobulated) Microcalcifications Taller-than-wide shape Rim calcifications with small extrusive soft-tissue component Evidence of extrathyroidal extension	Non-oval shape Irregular margins Microcalcifications Marked hypoechogenicity	Microcalcification Taller-than-wide shape Spiculated/microlobulated margins
Category	**Low-risk:** Cysts (fluid component >80%) Mostly cystic nodules with reverberating artifacts and not associated with suspicious US signs Isoechoic spongiform nodules, either confluent or with regular halo. *Risk of malignancy: 1%* *FNA >20 mm (selective)^a^*	**Benign:** Purely cystic nodules (no solid component) *Risk of malignancy: <1%* *FNA is not indicated*	**Benign (EU-TIRADS 2):** Pure/anechoic cysts; entirely spongiform nodules *Risk of malignancy: ≈ 0%* *FNA is not indicated*	**Benign:** Spongiform Partially cystic nodule with comet-tail artifact Pure cyst *Risk of malignancy: <1–3* *FNA ≥20 mm*
		**Very low suspicion:** Spongiform or partially cystic nodules without any of the US features defining low-, intermediate,- or high-suspicion patterns *Risk of malignancy: <3%* *FNA ≥20 mm or observation*	**Low-Risk (EU-TIRADS 3):** Oval shape, smooth margins, isoechoic, or hyperechoic, without any feature of high risk *Risk of malignancy: 2–4%* *FNA >20 mm*	**Low suspicion:** Partially cystic or isohyperechoic nodule without any of 3 suspicious US features* *Risk of malignancy: 3–15%* *FNA ≥15 mm*
		**Low suspicion:** Isoechoic or hyperechoic solid nodule, or partially cystic nodule with eccentric solid area without: microcalcifications, irregular margin, extrathyroidal extension, taller than wide shape *Risk of malignancy: 5–10% FNA ≥15 mm*		
	**Intermediate-risk:** Slightly hypoechoic (vs. thyroid tissue) or isoechoic nodules, with ovoid-to-round shape, smooth or ill-defined margins May be present: Intranodular vascularization Elevated stiffness at elastography, Macro or continuous rim calcifications Indeterminate hyperechoic spots *Risk of malignancy: 5–15%* *FNA: >20 mm*	**Intermediate suspicion:** Hypoechoic solid nodule with smooth margins without: microcalcifications, extrathyroidal extension or taller than wide shape *Risk of malignancy: 10–20%* *FNA ≥10 mm*	**Intermediate-Risk (EU-TIRADS 4):** Oval shape, smooth margins, mildly hypoechoic, without any feature of high risk *Risk of malignancy: 6–17%* *FNA >15 mm*	**Intermediate suspicion:** Solid hypoechoic nodule without any suspicious US feature or partially cystic or isohyperechoic nodule with any of the following: microcalcification, non-parallel orientation (taller-than-wide), spiculated/microlobulated margin *Risk of malignancy: 15–50%* *FNA ≥10 mm*
	**High-risk:** Nodules with ≥1 of the following: Marked hypoechogenicity (vs. prethyroid muscles) Spiculated or lobulated margins Microcalcifications Taller-than-wide shape (AP>TR) Extrathyroidal growth Pathologic adenopathy *Risk of malignancy: 50–90%^b^* *FNA ≥10 mm (5 mm, selective)^c^*	**High suspicion:** Solid hypoechoic nodule or solid hypoechoic component of partially cystic nodule with ≥1 of the following: Irregular margins (infiltrative, microlobulated) Microcalcifications Taller than wide shape Rim calcifications with small extrusive soft tissue Extrathyroidal extension *Risk of malignancy: >70–90%* *FNA ≥10 mm*	**High-Risk (EU-TIRADS 5):** Nodules with ≥1 of the following: Non-oval shape Irregular margins Microcalcifications Marked hypoechogenicity *Risk of malignancy: 26–87%* *FNA >10 mm*	**High suspicion:** Solid hypoechoic nodule with any of the following: Microcalcification Nonparallel orientation (taller-than-wide) Spiculated/microlobulated margin *Risk of malignancy: >60* *FNA ≥10 mm (>5 mm selective)*

a *Growing nodule, high-risk history, before surgery, or local therapies*.

b *In accordance with the presence of 1 or more suspicious findings*.

c *FNA is recommended for the following nodules: Subcapsular or paratracheal lesions; Suspicious lymph nodes or extrathyroid spread; Positive personal or family history of thyroid cancer: History of head and neck irradiation, coexistent suspicious clinical findings (e.g., dysphonia)*.

## Have Molecular Analysis an Added Value?

When thyroid nodules are evaluated with fine-needle aspiration biopsy (FNAB), in ~5–20% of cases it is not possible to discriminate between benign and malignant nodules because of an indeterminate cytology (78). According to The Bethesda System for Reporting Thyroid Cytopathology (79, 80) indeterminate cytology includes two different categories: atypical or follicular lesion (Bethesda III) and follicular neoplasm/suspicious for follicular (or Hürthle cell) neoplasm (Bethesda IV). The observed rates of cancer in these categories vary widely ranging from 6 to 48% for Bethesda III and 14 to 34% for Bethesda IV (81). This wide range of cancer risk, involves that diagnostic hemithyroidectomies are still performed in order to discriminate between benign and malignant nodules. Unfortunately, in 50–70% of patients with indeterminate cytology a diagnostic surgery is performed. Moreover, surgery exposes patients to surgical risks and in the event of malignant lesions, a second-stage surgery is often indicated with additional costs and risks for patients (82–85). Molecular tests using gene expression and/or mutational analysis, have been developed to reduce the need for diagnostic surgery for indeterminate (Bethesda III/IV) thyroid nodules (86). Several molecular tests have been proposed over the years, since different gene-mutation panels has been introduced (86), with reported NPV and PPV ranging from 56 to 100% and from 19 to 100%, respectively, and the most successful are the Thyroseq and the AFIRMA Gene Expression Classifier (GEC). The first version of Thyroseq included the 7 gene panel (BRAF, H-K-N-RAS, RET/PTC1-3, PAX8/PPARγ) (87) with a reported sensitivity of about 65% (87–89). Following versions migrated to the next generation sequencing platforms (NGS) and included a 13-gene panel (ThyroSeq v1) (90) and a 56-gene panel (ThyroSeq v2) with a significant increase in sensitivity and negative predictive value (NPV) (91, 92). The last version of Thyroseq, v3, Nikiforov and Baloch (92) is a targeted NGS test that evaluates point mutations, gene fusions, copy number alterations and abnormal gene expression in 112 thyroid cancer related genes. Using the last version of Thyroseq, a recent prospective and multicenter validation study (93) on 286 cytological indeterminate nodules submitted to surgery reported a 94% of sensitivity and 82% of specificity with a NPV of 97% and a positive predictive value (PPV) of 66%. These data may obviate diagnostic surgery in up to 61% of patients with indeterminate nodules. The AFIRMA GEC is a microarray based test with a proprietary algorithm able to differentiate benign from malignant nodules based on messenger RNA expression pattern. The sensitivity is approximately of 90%, but the specificity is lower (88, 94). Moreover, a significant site to site variability in the benign call rate (range 27–53%) and in the malignancy rate (range 15.6–70%) was reported in a follow-up multicenter study (95).Very recently, the AFIRMA Genomic Sequencing Classifier (GSC) replaced the original GEC. It is a RNA sequencing based test, including 12 classifiers composed of 10,196 genes and 7 additional components in order to exclude parathyroid lesions and medullary thyroid cancer, and includes the analysis of BRAFV600E mutations, RET/PTC1 or RET/PTC3 and of specific alterations typical of Hurtle cell lesions. Compared to GEC, the GSC has a better specificity and reduces the number of histological benign samples classified as suspicious. An initial validation study showed a 36% increase in specificity compared with the GEC with a reported sensitivity of about 91% (96). Harrell et al. (97) demonstrated that GSC is able to identify less indeterminate cytology nodules as suspicious when compared to GEC, suggesting that GSC further reduces surgery by improving in specificity. In a recent independent study, Endo et al. (98) compared GEC with GSC and demonstrated that GSC had a significant higher benign call rate (76.2 vs. 48.1%), PPV (60.0 vs. 33.3%), and specificity (94.3 vs. 61.4%) than GEC in both Bethesda III and Bethesda IV categories. In particular, benign call rate of GSC was significantly higher in nodules with Hürthle cell changes (88.8 vs. 25.7%). In summary, both ThyroSeq and AFIRMA have reached a high sensitivity and enough specificity to function as rule in and rule out tests. The main problem is the limited number of validation studies and the high costs that remain a limit in their worldwide utilization. Currently, there are no data to prefer a molecular test rather than another one, and long term outcome data are needed.

## Mini Invasive Treatments

Most benign thyroid nodules are asymptomatic, stable and do not require treatment, while large thyroid nodules may become responsible for pressure symptom, neck discomfort or cosmetic complaints thus resulting in decreased quality of life (99). Over the last two decades, non-surgical, minimally invasive US-guided techniques have been proposed for the treatment of symptomatic nodules. Minimally invasive procedures include percutaneous ethanol injection (PEI), laser thermal ablation (LTA), radiofrequency ablation (RFA), high intensity focused ultrasound (HIFU), and percutaneous microwave ablation (PMWA) (Table 2). PEI represents the first-line treatment for thyroid cysts and nodules with a predominant fluid component (100), while in solid nodules, LTA and RFA have proven to be very effective and safe in producing significant and stable reduction of nodule volume (101). Radiofrequency thermoablation consists in thermal ablation of the nodular tissue by exploiting the heat released by an energy source with consequent coagulation necrosis. The purpose of the treatment is to determine a volumetric reduction of the thyroid nodule, a condition that usually occurs in the weeks and months following the procedure as a consequence of the gradual replacement of the thyroid tissue with fibro-scar tissue and the procedure can be repeated after some time (102). Overall complication rate is low, about 3.5% (103). Some authors reported an higher difficulty of surgery after treatments, and exists the rare possibility of cancer spreading while treating patients with supposedly benign nodules (104). Radiofrequency thermoablation can be used for the treatment of benign nodular masses on cytological evaluation, which cause aesthetic alteration or compressive symptoms which cannot be treated surgically, for comorbidities or patient's preference. It is also recommended for the treatment of both pre-toxic and toxic nodules, when surgery or radioiodine are contraindicated or refused by the patient (3, 102, 105). Radiofrequency thermoablation has been proposed for papillary thyroid microcarcinoma and in cases of recurrence or loco-regional persistence of thyroid carcinoma when surgery is contraindicated or radiometabolic therapy has proved ineffective (18, 106). Some limitations still remain, such as the difficult to determine if cancer cells are fully eliminated even if ablation zones completely disappear on US and long-term outcomes (107). Another procedure based on the principles of hyperthermia is LTA that significantly reduce thyroid nodule volume as well as symptoms and cosmetic problems, due to coagulative necrosis into the target tissue (108, 109). A 3-year multicenter prospective randomized trial with LTA showed persistent volume reduction and local symptom improvement at 36 months after treatment (110). A systematic meta-analysis, comparing the efficacy of RFA and LTA for the treatment of benign thyroid nodules, concluded that both LTA and RFA are able to significantly decrease nodule volume, though RFA has a superior efficacy to LTA in nodule shrinkage despite minor number of treatment sessions (111). Only one study reported minor complications, as transient thyrotoxicosis and fever, after LTA (112) while no studies reported major complications such as voice change or hypothyroidism after either RFA or LA. It remains unclear if the different results are linked to the different energy delivered per ml of thyroid tissue, to the treatment time or technique. Finally, HIFU and PMWA are other promising forms of thermal ablation technique, but need further clinical testing. High intensity focused ultrasound (HIFU) have some advantages over other ablation techniques such as the ability to induce a focused thermal tissue destruction without needle puncture and seems to be less dependent on the skill of the operator. However, it produces thermal coagulation within a small volume and the ablation of a larger tissue volume may take an excessive period of time (113). The treatment efficacy (i.e., extent of nodule shrinkage at 6-month) in larger-sized benign thyroid nodules has been evaluated by Lang et al. in 63 nodules with a noticeably less efficacy for larger-sized nodules (114). HIFU is a safe treatment although transient side effects have been reported, such as pain, skin redness, mild subcutaneous swelling and transient vocal cord paralysis (115, 116). Percutaneous microwave ablation (PMWA) is a new technique that produce a rapid increase of the target tissue temperature through the rotation of molecules produced from microwave energy. Few studies analyzed the effectiveness of PMWA in the treatment of benign solid thyroid nodules. Liu et al. evaluated 474 benign thyroid nodules in 435 patients treated with PMWA showing a mean 90% decrease in volume at 1-year, with no major complications described (70). Another study by Yue et al. reported, in 110 patients treated with PMWA, a significant reduction at 1 year (ranging from 12.6 ± 15.1 to 3.2 ± 5.7 mL) (117). A retrospective, observational trial at a single institution compared the efficacy and safety of RFA in 40 patients, PMWA in 40 patients and HIFU in 14 patients with small nodules at 3 months after ablation. RFA showed a slightly better mean volume reduction of nodules (50%) than MWA (44%) and HIFU (48%). The study limitation is the short period of time (118). In conclusion, non-surgical minimally invasive approaches can be used to treat symptomatic or enlarging thyroid nodule and appear safe and effective. Currently, percutaneous ethanol injection (PEI) is recommended for symptomatic cystic or relapsing cystic lesions. Either laser thermal ablation (LTA) or radiofrequency ablation (RFA) can be used for symptomatic solid nodules. Microwave ablation (PMWA) or high intensity focused ultrasound (HIFU) are newer techniques with promising results that await further clinical evaluation.

**Table 2:** An overview of the non-surgical, Image-Guided, Minimally Invasive Therapy for thyroid nodules or recurrent thyroid cancer.

**Clinical Indication**		**Treatment**	
		**First line**	**Second Line**
Cysts or predominantly cystic benign thyroid nodules	Cystic nodules (>90% of fluid composition) Predominantly cystic nodules (51–90% of fluid component)	Us-guided percutaneous ethanol ablation (PEI)	Us-guided thermal ablation *RFA can be recommended as the next step in cases with incomplete resolved symptoms due to the residual solid component or recurrence following PEI*
Solid non-functioning (cold) benign thyroid nodules	Benign, non-functioning solid nodules with symptoms or cosmetic problems Benign, non-functioning solid nodules that progressively enlarge Benign multinodular goiter in patient who refuse or cannot undergo surgery	Thermal ablation (Radiofrequency ablation, laser ablation) Surgery	
Autonomously functioning thyroid nodules (AFTN)		Radioactive iodine (RAI) Surgery	Thermal ablation (Radiofrequency ablation, laser ablation)
Primary Thyroid Cancer Follicular neoplasm		Surgery	Thermal ablation *Who refuse surgery or who cannot undergo an operation, thermal ablations can be considered as an alternative. Radiofrequency ablation, laser ablation, and microwave ablation have been attempted for patients with papillary thyroid microcarcinoma (PTMC)*.
DTC patients with metastatic disease		Surgery TSH-suppressive thyroid hormone therapy for patients with stable or slowly progressive asymptomatic disease 131-I therapy for RAI-responsive disease	External beam radiation therapy Thermal ablation Systemic therapy with kinase inhibitors

## Discussion

In the era of precision medicine, the most important landmark remains the correct identification of malignant thyroid nodules. Newer and promising imaging techniques combined with the more accurate molecular examination will be able to reduce diagnostic uncertainty. Moreover, newer therapeutic options will be able to reduce, when possible, avoidable thyroidectomy. These approaches will allow the clinician to set up a tailored management, from diagnosis to treatment, of thyroid nodule disease, according to the patient's needs.

## Author Contributions

LF and MC contributed to conception and design of the review. DT, GG, MDS, MDM, MS, TR, and FM wrote the first draft of the manuscript. All authors wrote sections of the manuscript, contributed to manuscript revision and approved it for publication.

### Conflict of Interest

The authors declare that the research was conducted in the absence of any commercial or financial relationships that could be construed as a potential conflict of interest.

## References

[1] Gharib H Papini E. Thyroid nodules: clinical importance, assessment, and treatment. Endocrinol Metab Clin North Am. (2007) 36:707–35. 10.1016/j.ecl.2007.04.009 17673125

[2] Burman KD Wartofsky L. CLINICAL PRACTICE. Thyroid nodules. N Engl J Med. (2015) 373:2347–56. 10.1056/NEJMcp1415786 26650154

[3] Gharib H Papini E Garber JR Duick DS Harrell RM Hegedus L. American Association of Clinical Endocrinologists, American College of Endocrinology, and Associazione Medici Endocrinologi Medical Guidelines for Clinical Practice for the Diagnosis and Management of Thyroid Nodules−2016 Update. Endocr Pract. (2016) 22:622–39. 10.4158/EP161208.GL 27167915

[4] Smith-Bindman R Lebda P Feldstein VA Sellami D Goldstein RB Brasic N. Risk of thyroid cancer based on thyroid ultrasound imaging characteristics: results of a population-based study. JAMA Intern Med. (2013) 173:1788–96. 10.1001/jamainternmed.2013.9245 23978950 PMC3936789

[5] Nam-Goong IS Kim HY Gong G Lee HK Hong SJ Kim WB. Ultrasonography-guided fine-needle aspiration of thyroid incidentaloma: correlation with pathological findings. Clin Endocrinol. (2004) 60:21–8. 10.1046/j.1365-2265.2003.01912.x 14678283

[6] Durante C Grani G Lamartina L Filetti S Mandel SJ Cooper DS. The diagnosis and management of thyroid nodules: a review. JAMA. (2018) 319:914–24. 10.1001/jama.2018.0898 29509871

[7] Brito JP Ito Y Miyauchi A Tuttle RM. A clinical framework to facilitate risk stratification when considering an active surveillance alternative to immediate biopsy and surgery in papillary microcarcinoma. Thyroid. (2016) 26:144–9. 10.1089/thy.2015.0178 26414743 PMC4842944

[8] Russ G Bonnema SJ Erdogan MF Durante C Ngu R Leenhardt L. European thyroid association guidelines for ultrasound malignancy risk stratification of thyroid nodules in adults: the EU-TIRADS. Eur Thyroid J. (2017) 6:225–37. 10.1159/000478927 29167761 PMC5652895

[9] Tessler FN Middleton WD Grant EG Hoang JK Berland LL Teefey SA. ACR Thyroid Imaging, Reporting and Data System (TI-RADS): white paper of the ACR TI-RADS committee. J Am Coll Radiol. (2017) 14:587–95. 10.1016/j.jacr.2017.01.046 28372962

[10] Grant EG Tessler FN Hoang JK Langer JE Beland MD Berland LL. Thyroid ultrasound reporting lexicon: white paper of the ACR Thyroid Imaging, Reporting and Data System (TIRADS) committee. J Am Coll Radiol. (2015) 12(12 Pt A):1272–9. 10.1016/j.jacr.2015.07.011 26419308

[11] Brito JP Gionfriddo MR Al Nofal A Boehmer KR Leppin AL Reading C. The accuracy of thyroid nodule ultrasound to predict thyroid cancer: systematic review and meta-analysis. J Clin Endocrinol Metab. (2014) 99:1253–63. 10.1210/jc.2013-2928 24276450 PMC3973781

[12] Campanella P Ianni F Rota CA Corsello SM Pontecorvi A. Quantification of cancer risk of each clinical and ultrasonographic suspicious feature of thyroid nodules: a systematic review and meta-analysis. Eur J Endocrinol. (2014) 170:R203–11. 10.1530/EJE-13-0995 24536085

[13] Razavi SA Hadduck TA Sadigh G Dwamena BA. Comparative effectiveness of elastographic and B-mode ultrasound criteria for diagnostic discrimination of thyroid nodules: a meta-analysis. AJR Am J Roentgenol. (2013) 200:1317–26. 10.2214/AJR.12.9215 23701071

[14] Remonti LR Kramer CK Leitao CB Pinto LC Gross JL. Thyroid ultrasound features and risk of carcinoma: a systematic review and meta-analysis of observational studies. Thyroid. (2015) 25:538–50. 10.1089/thy.2014.0353 25747526 PMC4447137

[15] Frates MC Benson CB Charboneau JW Cibas ES Clark OH Coleman BG. Management of thyroid nodules detected at US: Society of Radiologists in Ultrasound consensus conference statement. Radiology. (2005) 237:794–800. 10.1148/radiol.2373050220 16304103

[16] Grani G D'Alessandri M Carbotta G Nesca A Del Sordo M Alessandrini S. Grey-scale analysis improves the ultrasonographic evaluation of thyroid nodules. Medicine. (2015) 94:e1129. 10.1097/MD.0000000000001129 26166117 PMC4504637

[17] Park SJ Park SH Choi YJ Kim DW Son EJ Lee HS. Interobserver variability and diagnostic performance in US assessment of thyroid nodule according to size. Ultraschall Med. (2012) 33:E186–90. 10.1055/s-0032-1325404 23108925

[18] Haugen BR Alexander EK Bible KC Doherty GM Mandel SJ Nikiforov YE. 2015 American thyroid association management guidelines for adult patients with thyroid nodules and differentiated thyroid cancer: the American thyroid association guidelines task force on thyroid nodules and differentiated thyroid cancer. Thyroid. (2016) 26:1–133. 10.1089/thy.2015.0020 26462967 PMC4739132

[19] Shin JH Baek JH Chung J Ha EJ Kim JH Lee YH. Ultrasonography diagnosis and imaging-based management of thyroid nodules: revised korean society of thyroid radiology consensus statement and recommendations. Korean J Radiol. (2016) 17:370–95. 10.3348/kjr.2016.17.3.370 27134526 PMC4842857

[20] Middleton WD Teefey SA Reading CC Langer JE Beland MD Szabunio MM. Multiinstitutional analysis of thyroid nodule risk stratification using the American college of radiology thyroid imaging reporting and data system. AJR Am J Roentgenol. (2017) 208:1331–41. 10.2214/AJR.16.17613 28402167

[21] Ha EJ Moon WJ Na DG Lee YH Choi N Kim SJ. A multicenter prospective validation study for the Korean thyroid imaging reporting and data system in patients with thyroid nodules. Korean J Radiol. (2016) 17:811–21. 10.3348/kjr.2016.17.5.811 27587972 PMC5007410

[22] Trimboli P Ngu R Royer B Giovanella L Bigorgne C Simo R. A multicentre validation study for the EU-TIRADS using histological diagnosis as a gold standard. Clin Endocrinol. (2019) 91:340–7. 10.1111/cen.13997 31002419

[23] Nam SJ Kwak JY Moon HJ Yoon JH Kim EK Koo JS. Large (>/=3cm) thyroid nodules with benign cytology: can Thyroid Imaging Reporting and Data System (TIRADS) help predict false-negative cytology? PLoS ONE. (2017) 12:e0186242. 10.1371/journal.pone.0186242 29023564 PMC5638398

[24] Mendes GF Garcia MR Falsarella PM Rahal A Cavalcante FA Junior Nery DR. Fine needle aspiration biopsy of thyroid nodule smaller than 1.0 cm: accuracy of TIRADS classification system in more than 1000 nodules. Br J Radiol. (2018) 91:20170642. 10.1259/bjr.20170642 29182368 PMC5965458

[25] Zheng Y Xu S Kang H Zhan W. A single-center retrospective validation study of the American College of radiology thyroid imaging reporting and data system. Ultrasound Q. (2018) 34:77–83. 10.1097/RUQ.0000000000000350 29596298

[26] Koseoglu Atilla FD Ozgen Saydam B Erarslan NA Diniz Unlu AG Yilmaz Yasar H Ozer M. Does the ACR TI-RADS scoring allow us to safely avoid unnecessary thyroid biopsy? single center analysis in a large cohort. Endocrine. (2018) 61:398–402. 10.1007/s12020-018-1620-6 29744655

[27] Hoang JK Middleton WD Farjat AE Langer JE Reading CC Teefey SA. Reduction in thyroid nodule biopsies and improved accuracy with American college of radiology thyroid imaging reporting and data system. Radiology. (2018) 287:185–93. 10.1148/radiol.2018172572 29498593

[28] Yoon JH Lee HS Kim EK Moon HJ Kwak JY. Malignancy risk stratification of thyroid nodules: comparison between the thyroid imaging reporting and data system and the 2014 American thyroid association management guidelines. Radiology. (2016) 278:917–24. 10.1148/radiol.2015150056 26348102

[29] Persichetti A Di Stasio E Guglielmi R Bizzarri G Taccogna S Misischi I. Predictive value of malignancy of thyroid nodule ultrasound classification systems: a prospective study. J Clin Endocrinol Metab. (2018) 103:1359–68. 10.1210/jc.2017-01708 29408952

[30] Maino F Forleo R Martinelli M Fralassi N Barbato F Pilli T. Prospective validation of ATA and ETA sonographic pattern risk of thyroid nodules selected for FNAC. J Clin Endocrinol Metab. (2018) 103:2362–8. 10.1210/jc.2018-00274 29672763

[31] Grani G Lamartina L Ascoli V Bosco D Biffoni M Giacomelli L. Reducing the number of unnecessary thyroid biopsies while improving diagnostic accuracy: toward the “right” TIRADS. J Clin Endocrinol Metab. (2019) 104:95–102. 10.1210/jc.2018-01674 30299457

[32] Grani G Lamartina L Cantisani V Maranghi M Lucia P Durante C. Interobserver agreement of various thyroid imaging reporting and data systems. Endocr Connect. (2018) 7:1–7. 10.1530/EC-17-0336 29196301 PMC5744624

[33] Valderrabano P McGettigan MJ Lam CA Khazai L Thompson ZJ Chung CH. Thyroid nodules with indeterminate cytology: utility of the american thyroid association sonographic patterns for cancer risk stratification. Thyroid. (2018) 28:1004–12. 10.1089/thy.2018.0085 29848195 PMC6916126

[34] Tang AL Falciglia M Yang H Mark JR Steward DL. Validation of American thyroid association ultrasound risk assessment of thyroid nodules selected for ultrasound fine-needle aspiration. Thyroid. (2017) 27:1077–82. 10.1089/thy.2016.0555 28657511

[35] Grani G Lamartina L Ascoli V Bosco D Nardi F D'Ambrosio F. Ultrasonography scoring systems can rule out malignancy in cytologically indeterminate thyroid nodules. Endocrine. (2017) 57:256–61. 10.1007/s12020-016-1148-6 27804016

[36] Sollini M Cozzi L Chiti A Kirienko M. Texture analysis and machine learning to characterize suspected thyroid nodules and differentiated thyroid cancer: where do we stand? Eur J Radiol. (2018) 99:1–8. 10.1016/j.ejrad.2017.12.004 29362138

[37] Yoo YJ Ha EJ Cho YJ Kim HL Han M Kang SY. Computer-aided diagnosis of thyroid nodules via ultrasonography: initial clinical experience. Korean J Radiol. (2018) 19:665–72. 10.3348/kjr.2018.19.4.665 29962872 PMC6005935

[38] Jeong EY Kim HL Ha EJ Park SY Cho YJ Han M. Computer-aided diagnosis system for thyroid nodules on ultrasonography: diagnostic performance and reproducibility based on the experience level of operators. Eur Radiol. (2019) 29:1978–85. 10.1007/s00330-018-5772-9 30350161

[39] Ko SY Lee JH Yoon JH Na H Hong E Han K. Deep convolutional neural network for the diagnosis of thyroid nodules on ultrasound. Head Neck. (2019) 41:885–91. 10.1002/hed.25415 30715773

[40] Rago T Santini F Scutari M Pinchera A Vitti P. Elastography: new developments in ultrasound for predicting malignancy in thyroid nodules. J Clin Endocrinol Metab. (2007) 92:2917–22. 10.1210/jc.2007-0641 17535993

[41] Asteria C Giovanardi A Pizzocaro A Cozzaglio L Morabito A Somalvico F. US-elastography in the differential diagnosis of benign and malignant thyroid nodules. Thyroid. (2008) 18:523–31. 10.1089/thy.2007.0323 18466077

[42] Ding J Cheng H Ning C Huang J Zhang Y. Quantitative measurement for thyroid cancer characterization based on elastography. J Ultrasound Med. (2011) 30:1259–66. 10.7863/jum.2011.30.9.1259 21876097

[43] Rago T Scutari M Santini F Loiacono V Piaggi P Di Coscio G Real-time elastosonography: useful tool for refining the presurgical diagnosis in thyroid nodules with indeterminate or nondiagnostic cytology. J Clin Endocrinol Metab. (2010) 95:5274–80. 10.1210/jc.2010-0901 20810572

[44] Rago T Vitti P. Role of thyroid ultrasound in the diagnostic evaluation of thyroid nodules. Best Pract Res Clin Endocrinol Metab. (2008) 22:913–28. 10.1016/j.beem.2008.09.016 19041822

[45] Shiina T Nightingale KR Palmeri ML Hall TJ Bamber JC Barr RG. WFUMB guidelines and recommendations for clinical use of ultrasound elastography: part 1: basic principles and terminology. Ultrasound Med Biol. (2015) 41:1126–47. 10.1016/j.ultrasmedbio.2015.03.009 25805059

[46] Kwak JY Kim EK. Ultrasound elastography for thyroid nodules: recent advances. Ultrasonography. (2014) 33:75–82. 10.14366/usg.13025 24936499 PMC4058985

[47] Sun CY Lei KR Liu BJ Bo XW Li XL He YP. Virtual touch tissue imaging and quantification (VTIQ) in the evaluation of thyroid nodules: the associated factors leading to misdiagnosis. Sci Rep. (2017) 7:41958. 10.1038/srep41958 28157195 PMC5291218

[48] Cantisani V Lodise P Grazhdani H Mancuso E Maggini E Di Rocco G. Ultrasound elastography in the evaluation of thyroid pathology. Current status. Eur J Radiol. (2014) 83:420–8. 10.1016/j.ejrad.2013.05.008 23763859

[49] Wang J Li P Sun L Sun Y Fang S Liu X. Diagnostic value of strain ratio measurement in differential diagnosis of thyroid nodules coexisted with Hashimoto thyroiditis. Int J Clin Exp Med. (2015) 8:6420–6. 26131268 PMC4483862

[50] Cosgrove D Barr R Bojunga J Cantisani V Chammas MC Dighe M. WFUMB guidelines and recommendations on the clinical use of ultrasound elastography: part 4. thyroid. Ultrasound Med Biol. (2017) 43:4–26. 10.1016/j.ultrasmedbio.2016.06.022 27570210

[51] Veyrieres JB Albarel F Lombard JV Berbis J Sebag F Oliver C. A threshold value in Shear Wave elastography to rule out malignant thyroid nodules: a reality? Eur J Radiol. (2012) 81:3965–72. 10.1016/j.ejrad.2012.09.002 23031543

[52] Cho YJ Ha EJ Han M Choi JW. US elastography using carotid artery pulsation may increase the diagnostic accuracy for thyroid nodules with US-pathology discordance. Ultrasound Med Biol. (2017) 43:1587–95. 10.1016/j.ultrasmedbio.2017.04.007 28528019

[53] Zhang YF Xu HX He Y Liu C Guo LH Liu LN. Virtual touch tissue quantification of acoustic radiation force impulse: a new ultrasound elastic imaging in the diagnosis of thyroid nodules. PLoS ONE. (2012) 7:e49094. 10.1371/journal.pone.0049094 23152855 PMC3496737

[54] Zhou H Zhou XL Xu HX Li DD Liu BJ Zhang YF. Virtual touch tissue imaging and quantification in the evaluation of thyroid nodules. J Ultrasound Med. (2017) 36:251–60. 10.7863/ultra.15.12070 27914177

[55] Ragazzoni F Deandrea M Mormile A Ramunni MJ Garino F Magliona G. High diagnostic accuracy and interobserver reliability of real-time elastography in the evaluation of thyroid nodules. Ultrasound Med Biol. (2012) 38:1154–62. 10.1016/j.ultrasmedbio.2012.02.025 22542262

[56] Cantisani V Grazhdani H Ricci P Mortele K Di Segni M D'Andrea V. Q-elastosonography of solid thyroid nodules: assessment of diagnostic efficacy and interobserver variability in a large patient cohort. Eur Radiol. (2014) 24:143–50. 10.1007/s00330-013-2991-y 23979108

[57] Hofauer B Mansour N Heiser C Wirth M Strassen U Loeffelbein D. Reproducibility of acoustic radiation force impulse imaging in thyroid and salivary glands with experienced and inexperienced examiners. Ultrasound Med Biol. (2016) 42:2545–52. 10.1016/j.ultrasmedbio.2016.06.019 27475926

[58] Park SH Kim SJ Kim EK Kim MJ Son EJ Kwak JY. Interobserver agreement in assessing the sonographic and elastographic features of malignant thyroid nodules. AJR Am J Roentgenol. (2009) 193:W416–23. 10.2214/AJR.09.2541 19843721

[59] Lim DJ Luo S Kim MH Ko SH Kim Y. Interobserver agreement and intraobserver reproducibility in thyroid ultrasound elastography. AJR Am J Roentgenol. (2012) 198:896–901. 10.2214/AJR.11.7009 22451558

[60] Kim JK Baek JH Lee JH Kim JL Ha EJ Kim TY. Ultrasound elastography for thyroid nodules: a reliable study? Ultrasound Med Biol. (2012) 38:1508–13. 10.1016/j.ultrasmedbio.2012.05.017 22766122

[61] Friedrich-Rust M Romenski O Meyer G Dauth N Holzer K Grunwald F. Acoustic Radiation Force Impulse-Imaging for the evaluation of the thyroid gland: a limited patient feasibility study. Ultrasonics. (2012) 52:69–74. 10.1016/j.ultras.2011.06.012 21788057

[62] Calvete AC Rodriguez JM de Dios Berna-Mestre J Rios A Abellan-Rivero D Reus M. Interobserver agreement for thyroid elastography: value of the quality factor. J Ultrasound Med. (2013) 32:495–504. 10.7863/jum.2013.32.3.495 23443190

[63] Grazhdani H Cantisani V Lodise P Di Rocco G Proietto MC Fioravanti E. Prospective evaluation of acoustic radiation force impulse technology in the differentiation of thyroid nodules: accuracy and interobserver variability assessment. J Ultrasound. (2014) 17:13–20. 10.1007/s40477-013-0062-5 24616747 PMC3945197

[64] Swan KZ Nielsen VE Bibby BM Bonnema SJ. Is the reproducibility of shear wave elastography of thyroid nodules high enough for clinical use? A methodological study. Clin Endocrinol. (2017) 86:606–13. 10.1111/cen.13295 28002625

[65] Andrioli M Scacchi M Carzaniga C Vitale G Moro M Poggi L. Thyroid nodules in acromegaly: the role of elastography. J Ultrasound. (2010) 13:90–7. 10.1016/j.jus.2010.09.008 23396892 PMC3553338

[66] Andrioli M Valcavi R. The peculiar ultrasonographic and elastographic features of thyroid nodules after treatment with laser or radiofrequency: similarities and differences. Endocrine. (2014) 47:967–8. 10.1007/s12020-014-0241-y 24664362

[67] Andrioli M Trimboli P Amendola S Valabrega S Fukunari N Mirella M. Elastographic presentation of medullary thyroid carcinoma. Endocrine. (2014) 45:153–5. 10.1007/s12020-013-0062-4 24065313

[68] Magri F Chytiris S Capelli V Alessi S Nalon E Rotondi M. Shear wave elastography in the diagnosis of thyroid nodules: feasibility in the case of coexistent chronic autoimmune Hashimoto's thyroiditis. Clin Endocrinol. (2012) 76:137–41. 10.1111/j.1365-2265.2011.04170.x 21740455

[69] Li Y Wang Y Wu Q Hu B. Papillary thyroid microcarcinoma co-exists with Hashimoto's thyroiditis: Is strain elastography still useful? Ultrasonics. (2016) 68:127–33. 10.1016/j.ultras.2016.02.013 26945905

[70] Liu YJ Qian LX Liu D Zhao JF. Ultrasound-guided microwave ablation in the treatment of benign thyroid nodules in 435 patients. Exp Biol Med. (2017) 242:1515–23. 10.1177/1535370217727477 28847173 PMC5648295

[71] Cappelli C Pirola I Gandossi E Formenti A Agosti B Castellano M. Elastography evaluation of benign thyroid nodules in patients affected by hashimoto's thyroiditis. Int J Endocrinol. (2015) 2015:367054. 10.1155/2015/367054 26273296 PMC4530237

[72] Cosgrove D Piscaglia F Bamber J Bojunga J Correas JM Gilja OH. EFSUMB guidelines and recommendations on the clinical use of ultrasound elastography. Part 2: Clinical applications. Ultraschall Med. (2013) 34:238–53. 10.1055/s-0033-1335375 23605169

[73] Moon HJ Sung JM Kim EK Yoon JH Youk JH Kwak JY. Diagnostic performance of gray-scale US and elastography in solid thyroid nodules. Radiology. (2012) 262:1002–13. 10.1148/radiol.11110839 22357900

[74] Trimboli P Guglielmi R Monti S Misischi I Graziano F Nasrollah N. Ultrasound sensitivity for thyroid malignancy is increased by real-time elastography: a prospective multicenter study. J Clin Endocrinol Metab. (2012) 97:4524–30. 10.1210/jc.2012-2951 23066117

[75] Cappelli C Pirola I Gandossi E Agosti B Cimino E Casella C. Real-time elastography: a useful tool for predicting malignancy in thyroid nodules with nondiagnostic cytologic findings. J Ultrasound Med. (2012) 31:1777–82. 10.7863/jum.2012.31.11.1777 23091248

[76] Yoon JH Moon HJ Kim EK Kwak JY Inadequate cytology in thyroid nodules: should we repeat aspiration or follow-up? Ann Surg Oncol. (2011) 18:1282–9. 10.1245/s10434-011-1605-7 21331807

[77] Trimboli P Treglia G Sadeghi R Romanelli F Giovanella L. Reliability of real-time elastography to diagnose thyroid nodules previously read at FNAC as indeterminate: a meta-analysis. Endocrine. (2015) 50:335–43. 10.1007/s12020-014-0510-9 25534701

[78] Baloch ZW Fleisher S LiVolsi VA Gupta PK. Diagnosis of “follicular neoplasm”: a gray zone in thyroid fine-needle aspiration cytology. Diagn Cytopathol. (2002) 26:41–4. 10.1002/dc.10043 11782086

[79] Baloch ZW LiVolsi VA Asa SL Rosai J Merino MJ Randolph G. Diagnostic terminology and morphologic criteria for cytologic diagnosis of thyroid lesions: a synopsis of the National Cancer Institute Thyroid Fine-Needle Aspiration State of the Science Conference. Diagn Cytopathol. (2008) 36:425–37. 10.1002/dc.20830 18478609

[80] Cibas ES Ali SZ. The 2017 bethesda system for reporting thyroid cytopathology. Thyroid. (2017) 27:1341–6. 10.1089/thy.2017.0500 29091573

[81] Bongiovanni M Spitale A Faquin WC Mazzucchelli L Baloch ZW. The bethesda system for reporting thyroid cytopathology: a meta-analysis. Acta Cytol. (2012) 56:333–9. 10.1159/000339959 22846422

[82] Vriens D Adang EM Netea-Maier RT Smit JW de Wilt JH Oyen WJ. Cost-effectiveness of FDG-PET/CT for cytologically indeterminate thyroid nodules: a decision analytic approach. J Clin Endocrinol Metab. (2014) 99:3263–74. 10.1210/jc.2013-3483 24873995

[83] McHenry CR Slusarczyk SJ. Hypothyroidisim following hemithyroidectomy: incidence, risk factors, and management. Surgery. (2000) 128:994–8. 10.1067/msy.2000.110242 11114634

[84] Rosato L Avenia N Bernante P De Palma M Gulino G Nasi PG. Complications of thyroid surgery: analysis of a multicentric study on 14,934 patients operated on in Italy over 5 years. World J Surg. (2004) 28:271–6. 10.1007/s00268-003-6903-1 14961204

[85] Jeannon JP Orabi AA Bruch GA Abdalsalam HA Simo R. Diagnosis of recurrent laryngeal nerve palsy after thyroidectomy: a systematic review. Int J Clin Pract. (2009) 63:624–9. 10.1111/j.1742-1241.2008.01875.x 19335706

[86] Paschke R Cantara S Crescenzi A Jarzab B Musholt TJ Sobrinho Simoes M. European thyroid association guidelines regarding thyroid nodule molecular fine-needle aspiration cytology diagnostics. Eur Thyroid J. (2017) 6:115–29. 10.1159/000468519 28785538 PMC5527175

[87] Nikiforov YE Ohori NP Hodak SP Carty SE LeBeau SO Ferris RL. Impact of mutational testing on the diagnosis and management of patients with cytologically indeterminate thyroid nodules: a prospective analysis of 1056 FNA samples. J Clin Endocrinol Metab. (2011) 96:3390–7. 10.1210/jc.2011-1469 21880806 PMC3205883

[88] Alexander EK Kennedy GC Baloch ZW Cibas ES Chudova D Diggans J. Preoperative diagnosis of benign thyroid nodules with indeterminate cytology. N Engl J Med. (2012) 367:705–15. 10.1056/NEJMoa1203208 22731672

[89] Nikiforov YE Steward DL Robinson-Smith TM Haugen BR Klopper JP Zhu Z. Molecular testing for mutations in improving the fine-needle aspiration diagnosis of thyroid nodules. J Clin Endocrinol Metab. (2009) 94:2092–8. 10.1210/jc.2009-0247 19318445

[90] Nikiforova MN Wald AI Roy S Durso MB Nikiforov YE. Targeted next-generation sequencing panel (ThyroSeq) for detection of mutations in thyroid cancer. J Clin Endocrinol Metab. (2013) 98:E1852–60. 10.1210/jc.2013-2292 23979959 PMC3816258

[91] Nikiforov YE Carty SE Chiosea SI Coyne C Duvvuri U Ferris RL. Highly accurate diagnosis of cancer in thyroid nodules with follicular neoplasm/suspicious for a follicular neoplasm cytology by ThyroSeq v2 next-generation sequencing assay. Cancer. (2014) 120:3627–34. 10.1002/cncr.29038 25209362 PMC7737376

[92] Nikiforov YE Baloch ZW. Clinical validation of the ThyroSeq v3 genomic classifier in thyroid nodules with indeterminate FNA cytology. Cancer Cytopathol. (2019) 127:225–30. 10.1002/cncy.22112 30811896 PMC6519348

[93] Steward DL Carty SE Sippel RS Yang SP Sosa JA Sipos JA. Performance of a multigene genomic classifier in thyroid nodules with indeterminate cytology: a prospective blinded multicenter study. JAMA Oncol. (2019) 5:204–12. 10.1001/jamaoncol.2018.4616 30419129 PMC6439562

[94] Vargas-Salas S Martinez JR Urra S Dominguez JM Mena N Uslar T. Genetic testing for indeterminate thyroid cytology: review and meta-analysis. Endocr Relat Cancer. (2018) 25:R163–77. 10.1530/ERC-17-0405 29255094 PMC5799829

[95] Alexander EK Schorr M Klopper J Kim C Sipos J Nabhan F. Multicenter clinical experience with the Afirma gene expression classifier. J Clin Endocrinol Metab. (2014) 99:119–25. 10.1210/jc.2013-2482 24152684

[96] Patel KN Angell TE Babiarz J Barth NM Blevins T Duh QY. Performance of a genomic sequencing classifier for the preoperative diagnosis of cytologically indeterminate thyroid nodules. JAMA Surg. (2018) 153:817–24. 10.1001/jamasurg.2018.1153 29799911 PMC6583881

[97] Harrell RM Eyerly-Webb SA Golding AC Edwards CM Bimston DN. Statistical comparison of afirma gsc and afirma gec outcomes in a community endocrine surgical practice: early findings. Endocr Pract. (2019) 25:161–4. 10.4158/EP-2018-0395 30383497

[98] Endo M Nabhan F Porter K Roll K Shirley LA Azaryan I. Afirma gene sequencing classifier compared with gene expression classifier in indeterminate thyroid nodules. Thyroid. (2019) 29:1115–24. 10.1089/thy.2018.0733 31154940 PMC7141558

[99] Cesareo R Palermo A Pasqualini V Cianni R Gaspa G Manfrini S. Radiofrequency ablation for the management of thyroid nodules: A critical appraisal of the literature. Clin Endocrinol (Oxf). (2017) 87:639–48. 10.1111/cen.13422 28718950

[100] Baek JH Ha EJ Choi YJ Sung JY Kim JK Shong YK. Radiofrequency versus ethanol ablation for treating predominantly cystic thyroid nodules: a randomized clinical trial. Korean J Radiol. (2015) 16:1332–40. 10.3348/kjr.2015.16.6.1332 26576124 PMC4644756

[101] Papini E Gugliemi R Pacella CM. Laser, radiofrequency, and ethanol ablation for the management of thyroid nodules. Curr Opin Endocrinol Diabetes Obes. (2016) 23:400–6. 10.1097/MED.0000000000000282 27504993

[102] Papini E Pacella CM Solbiati LA Achille G Barbaro D Bernardi S. Minimally-invasive treatments for benign thyroid nodules: a Delphi-based consensus statement from the Italian minimally-invasive treatments of the thyroid (MITT) group. Int J Hyperthermia. (2019) 36:376–82. 10.1080/02656736.2019.1575482 30909759

[103] Kim C Lee JH Choi YJ Kim WB Sung TY Baek JH. Complications encountered in ultrasonography-guided radiofrequency ablation of benign thyroid nodules and recurrent thyroid cancers. Eur Radiol. (2017) 27:3128–37. 10.1007/s00330-016-4690-y 27975148

[104] Oddo S Spina B Vellone VG Giusti M. A case of thyroid cancer on the track of the radiofrequency electrode 30 months after percutaneous ablation. J Endocrinol Invest. (2017) 40:101–2. 10.1007/s40618-016-0527-4 27531170

[105] Garberoglio R Aliberti C Appetecchia M Attard M Boccuzzi G Boraso F. Radiofrequency ablation for thyroid nodules: which indications? The first Italian opinion statement. J Ultrasound. (2015) 18:423–30. 10.1007/s40477-015-0169-y 26550079 PMC4630279

[106] Zhang M Luo Y Zhang Y Tang J. Efficacy and safety of ultrasound-guided radiofrequency ablation for treating low-risk papillary thyroid microcarcinoma: a prospective study. Thyroid. (2016) 26:1581–7. 10.1089/thy.2015.0471 27445090

[107] Kim JH Baek JH Sung JY Min HS Kim KW Hah JH. Radiofrequency ablation of low-risk small papillary thyroidcarcinoma: preliminary results for patients ineligible for surgery. Int J Hyperthermia. (2017) 33:212–9. 10.1080/02656736.2016.1230893 27590679

[108] Dossing H Bennedbaek FN Karstrup S Hegedus L. Benign solitary solid cold thyroid nodules: US-guided interstitial laser photocoagulation–initial experience. Radiology. (2002) 225:53–7. 10.1148/radiol.2251011042 12354983

[109] Pacella CM Mauri G Achille G Barbaro D Bizzarri G De Feo P. Outcomes and risk factors for complications of laser ablation for thyroid nodules: a multicenter study on 1531 patients. J Clin Endocrinol Metab. (2015) 100:3903–10. 10.1210/jc.2015-1964 26274342

[110] Papini E Rago T Gambelunghe G Valcavi R Bizzarri G Vitti P. Long-term efficacy of ultrasound-guided laser ablation for benign solid thyroid nodules. Results of a three-year multicenter prospective randomized trial. J Clin Endocrinol Metab. (2014) 99:3653–9. 10.1210/jc.2014-1826 25050903

[111] Ha EJ Baek JH Kim KW Pyo J Lee JH Baek SH. Comparative efficacy of radiofrequency and laser ablation for the treatment of benign thyroid nodules: systematic review including traditional pooling and bayesian network meta-analysis. J Clin Endocrinol Metab. (2015) 100:1903–11. 10.1210/jc.2014-4077 25695887

[112] Gambelunghe G Fatone C Ranchelli A Fanelli C Lucidi P Cavaliere A. A randomized controlled trial to evaluate the efficacy of ultrasound-guided laser photocoagulation for treatment of benign thyroid nodules. J Endocrinol Invest. (2006) 29:RC23–6. 10.1007/BF03347368 17114905

[113] Ebbini ES ter Haar G. Ultrasound-guided therapeutic focused ultrasound: current status and future directions. Int J Hyperthermia. (2015) 31:77–89. 10.3109/02656736.2014.995238 25614047

[114] Lang BH Woo YC Chiu KW. Single-session high-intensity focused ultrasound treatment in large-sized benign thyroid nodules. Thyroid. (2017) 27:714–21. 10.1089/thy.2016.0664 28326895

[115] Ambrosini CE Cianferotti L Picone A Torregrossa L Segnini G Frustaci G. High-intensity focused ultrasound as an alternative to the surgical approach in primary hyperparathyroidism: a preliminary experience. J Endocrinol Invest. (2011) 34:655–9. 10.1007/BF03345404 22156903

[116] Lang BH Wu ALH. High intensity focused ultrasound (HIFU) ablation of benign thyroid nodules - a systematic review. J Ther Ultrasound. (2017) 5:11. 10.1186/s40349-017-0091-1 28523127 PMC5434558

[117] Yue W Wang S Wang B Xu Q Yu S Yonglin Z. Ultrasound guided percutaneous microwave ablation of benign thyroid nodules: safety and imaging follow-up in 222 patients. Eur J Radiol. (2013) 82:e11–6. 10.1016/j.ejrad.2012.07.020 22940229

[118] Korkusuz Y Groner D Raczynski N Relin O Kingeter Y Grunwald F. Thermal ablation of thyroid nodules: are radiofrequency ablation, microwave ablation and high intensity focused ultrasound equally safe and effective methods? Eur Radiol. (2018) 28:929–35. 10.1007/s00330-017-5039-x 28894936

